# The role of HIF-1 in up-regulating MICA expression on human renal proximal tubular epithelial cells during hypoxia/reoxygenation

**DOI:** 10.1186/1471-2121-11-91

**Published:** 2010-11-23

**Authors:** Lei Luo, Jun Lu, Liang Wei, Dan Long, Jia Y Guo, Juan Shan, Fu S Li, Ping Y Lu, Ping Y Li, Li Feng

**Affiliations:** 1 Key Laboratory of Transplant Engineering and Immunology of Health Ministry of China; 2 Institute of Clinical Medicine, West China Hospital, Sichuan University (No.37 Guo Xue Xiang), Chengdu, 610041, China; 3 Chinese Evidence-Based Medicine Center, West China Hospital, Sichuan University (No.37 Guo Xue Xiang), Chengdu, 610041, China; 4 Transplantation Institute, West China Hospital, Sichuan University (No.37 Guo Xue Xiang), Chengdu, 610041, China

## Abstract

**Background:**

Human major histocompatibility complex class I-related chain A (MICA) plays a dual role in adaptive and innate immune responses. Increasing evidence demonstrates that MICA is closely correlated with acute and chronic kidney allograft rejection. Therefore, understanding the activation mechanisms of MICA is important in kidney transplantation. We previously demonstrated that ischemia/reperfusion injury (IRI) could up-regulate MICA expression on mouse kidney allografts. Since hypoxia-inducible factor-1 (HIF-1) is the master regulator of cellular adaptive responses to hypoxia during IRI, here we investigate whether HIF-1 could up-regulate MICA expression and its influence on NK cell cytotoxicity.

**Results:**

We find that HIF-1alpha plays an important role in up-regulating MICA expression, inducing IFNgamma secretion and NK cell cytotoxicity during hypoxia/reoxygenation. First, we generated a HIF-1alphaDELTAODD-expressing adenovirus to stably and functionally express HIF-1alpha in human renal proximal tubular epithelial (HK-2) cells under normoxia conditions. HIF-1alpha over-expression in HK-2 cells induces MICA expression and enhances NK cell cytotoxic activity towards cells that express HIF-1alpha. Second, we used a hypoxia/reoxygenation cell model to simulate IRI in vitro and found that the suppression of HIF-1alpha by RNAi induces down-regulation of MICA expression and inhibits NK cytotoxicity. In antibody blocking experiments, an anti-MICA mAb was able to down-regulate NK cell cytotoxic activity towards HK-2 cells that over-expressed HIF-1alpha. Moreover, when NK cells were co-cultured with the HK-2 cells expressing MICA, which was up-regulated by over-expression of HIF-1alpha, there was a significant increase in the secretion of IFNgamma. In the presence of the blocking MICA mAb, IFNgamma secretion was significantly decreased.

**Conclusions:**

These results demonstrate that hypoxia/reoxygenation-promoted MICA expression on HK-2 cells is through a HIF-1 pathway. The increased IFNgamma secretion and enhanced NK cell cytotoxicity was mainly due to the surface expression of MICA induced by over-expression of HIF-1alpha. This study enhances our understanding of MICA activation mechanisms during kidney transplantation and provides insights into how IRI can influence transplant outcome. Moreover, these findings might be also important for developing strategies to reduce the effect of MICA in kidney transplant outcomes in the future.

## Background

Since there are strong effects of the HLA antigens in transplant rejection, the role of non-HLA antigens in transplant rejection has not received much attention. However, in the past few years, there has been an increasing number of reports that kidney and heart transplants undergo acute or chronic rejection even with good HLA matches [1-5], suggesting that non-HLA antigens might also lead to graft loss.

The non-classical HLA molecule, human major histocompatibility complex class I-related chain A (MICA), is a functional gene located 46.4 kilobases centromeric to HLA-B and encodes a 62-kd cell surface glycoprotein, which has a molecular structure similar to class I HLA, but not associated with β2-microglobulin [6]. MICA is expressed on several cell types including endothelial cells, dendritic cells, fibroblasts and epithelial cells, but not on lymphocytes. It acts as a ligand for the immunostimulatory C-type lectin-like receptor NKG2 D, which is expressed on most natural killer (NK) cells and CD8+ T cells [6,7]. Since human NKG2 D is an activating receptor on NK cells [6], an increase of NKG2 D ligand (such as MICA) expression could enhance antigen specific CTL-mediated cytotoxicity by activating NK cells [8]. Moreover, MICA antigen expressed in the allograft could induce the generation of anti-MICA antibodies, which can kill cells in the presence of serum complement [9]. Thus, MICA plays a dual role in adaptive and innate immune responses and may affect the outcomes of solid organ transplantation. Many clinical studies have shown that the presence of MICA on kidney or heart transplant samples after transplantation is associated with acute and chronic allograft rejection [1,3,10,11]. Therefore, attempts to understand the activation mechanisms of MICA is receiving more and more attention in the solid organ transplantation setting.

It appears that MICA expression is up-regulated in tissues subjected to stress or injury [12]. Our previous studies showed that ischemia/reperfusion injury (IRI) could up-regulate MICA expression on mouse kidney and heart [13,14]. We also noticed that the accumulation of HIF-1alpha up-regulates MICA expression on human cardiomyocytes during hypoxia/reoxygenation [15]. It is possible that the expression of MICA in human kidney grafts could be also be induced by IRI.

Renal IRI is an inevitable process during transplantation. Hypoxia-inducible factor-1 (HIF-1) is the master regulator of cellular adaptive responses to hypoxia during IRI [16], which may activate the transcription of >100 genes crucial for adaptation to hypoxia [17]. It is a heterodimer consisting of an alpha-subunit (HIF-1alpha) and a β-subunit (HIF-1β), which belong to the basic helix-loop-helix (bHLH) family and PER-ARNT-SIM (PAS) domain-containing transcription factors [18]. While HIF-1β protein is constitutively present [19], there is a unique O_2_-dependent degradation domain (ODD) in HIF-1alpha, which leads to its degradation under normoxia conditions [20,21]. In contrast, HIF-1alpha is stabilized under hypoxic conditions, and combines with HIF-1β, where it induces physiological responses to hypoxia by binding to the hypoxia response element in hypoxia responsive genes, activating transcription [22]. Moreover, HIF-1alpha protein is constitutively active when the ODD domain is deleted (HIF-1alphaDELTAODD, deletion of aa. 401-603) [23,24]. In this study, we used a HIF-1alphaDELTAODD-expressing adenovirus to see whether HIF-1alpha, as a single factor in normoxia, could up-regulate MICA expression on the human renal proximal tubular epithelial cell line (HK-2) as it is known to do in primary human cardiomyocytes (HCMs) [15].

## Results

### Ad.CMV.HIF-1alphaDELTAODD mediated stable HIF-1 transcriptional activity in normoxia on HK-2 cells

**Figure 1:**
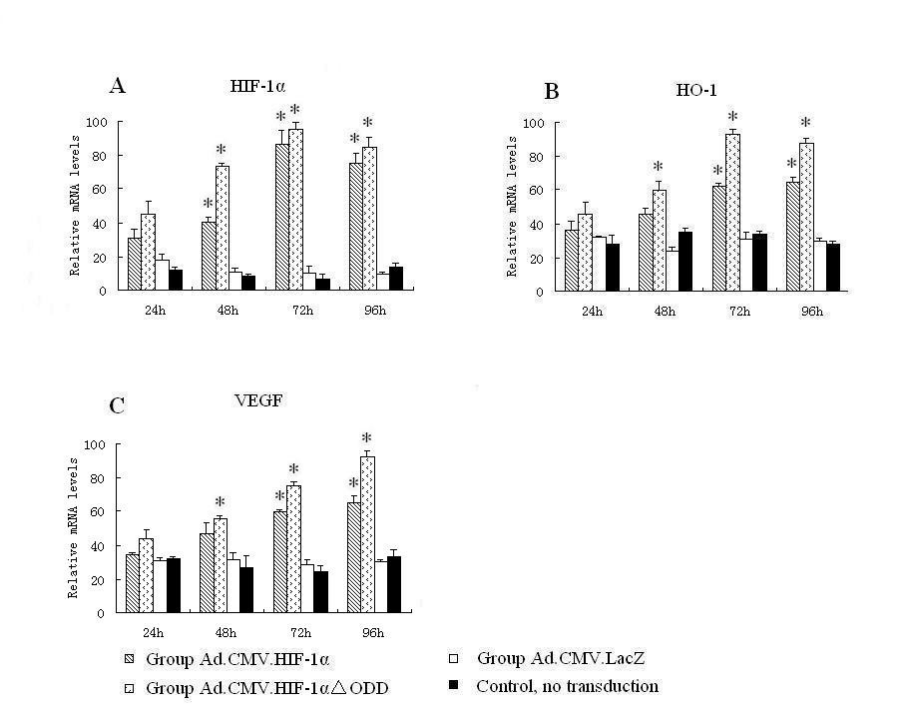
**Adenoviral mediated over-expression of HIF-1alpha and HIF-1alphaDELTAODD can up-regulate the transcription of VEGF and HO-1 in HK-2 cells in normoxia**. The relative mRNA levels of HIF-1alpha, HO-1 and VEGF were determined by real time PCR and normalized to the housekeeping gene β-actin. (A) Relative mRNA levels of HIF-1alpha. (B) Relative mRNA levels of HO-1. (C) Relative mRNA levels of VEGF. Values are shown as means ± SD from three independent experiments. *indicates a significant difference compared with control group (p < 0.05).

**Figure 2:**
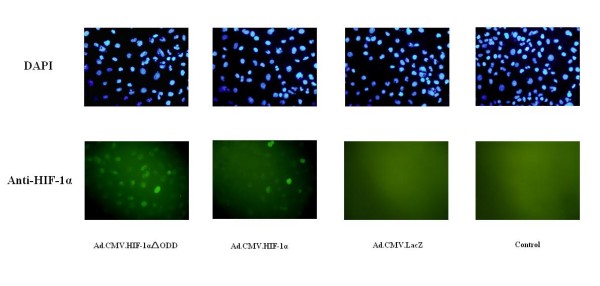
**Immunofluorescence studies of HIF-1alpha expression on HK-2 cells 96 h after adenoviral transduction**. DAPI staining was used to determine the number of nuclei. Anti-HIF-1alpha staining was used to determine the HIF-1alpha expression. Original magnification: ×200.

The adenovirus expressing HIF-1alphaDELTAODD was generated for stable HIF-1alpha expression in normoxia. HK-2 cells were collected for quantitative real-time PCR analysis and immunofluorescence studies at 24, 48, 72 and 96 h after transduction. Quantitative real-time PCR results showed that groups, which were transduced with Ad.CMV.HIF-1alphaDELTAODD or Ad.CMV.HIF-1alpha, had nearly 4--6 times higher HIF-1alpha mRNA levels compared with the group transduced with Ad.CMV.LacZ and the control group 48 h after transduction (Figure 1A). In addition, it was already known that HIF-1 can up-regulate the expression of VEGF [25] and HO-1 [26], and we demonstrated that VEGF and HO-1 mRNA levels increased significantly at 72 h and 96 h compared with the Ad.CMV.LacZ and control groups (Figure 1B and Figure 1C), suggesting that in HK-2 cells, HIF-1alpha without the ODD domain was transcriptionally active, like HIF-1alpha. Furthermore, the Ad.CMV.HIF-1alphaDELTAODD group had higher VEGF and HO-1 mRNA expression levels than the Ad.CMV.HIF-1alpha group, indicating that compared with HIF-1alpha, HIF-1alphaDELTAODD was functionally and steadily expressed regardless of the oxygen tension in normoxia. HIF-1alpha expression was virtually undetectable by immunofluorescence 48 h after transduction (data not shown). Increases in HIF-1alpha expression were detected in the groups transduced with Ad.CMV.HIF-1alphaDELTAODD and Ad.CMV.HIF-1alpha 72 and 96 h later, and HIF-1alpha expression was higher in the Ad.CMV.HIF-1alphaDELTAODD group. (Figure 2) Together, this suggests that Ad.CMV.HIF-1alphaDELTAODD could mediate stable HIF-1 transcriptional activity in normoxia in HK-2 cells.

### Expression of MICA increased in a HIF-1alpha dependent Manner in HK-2 cells

**Figure 3:**
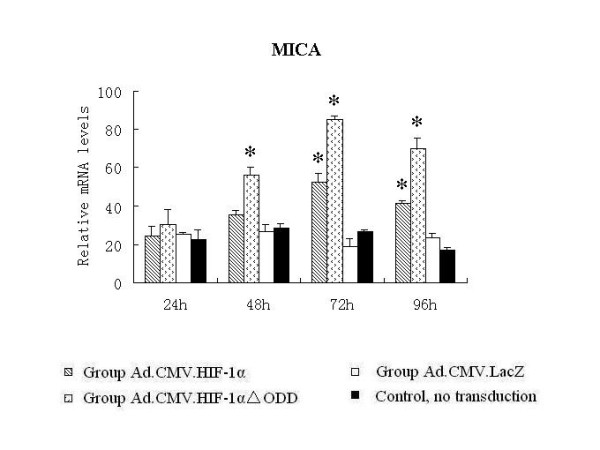
**Adenoviral mediated overexpression of HIF-1alpha and HIF-1alphaDELTAODD can up-regulate the transcription of MICA on HK-2 cells in normoxia**. The relative mRNA levels were determined by real-time PCR and normalized to the housekeeping gene β-actin. Values are shown as means ± SD from three independent experiments. *indicates significant difference compared with the control group (p < 0.05).

**Figure 4:**
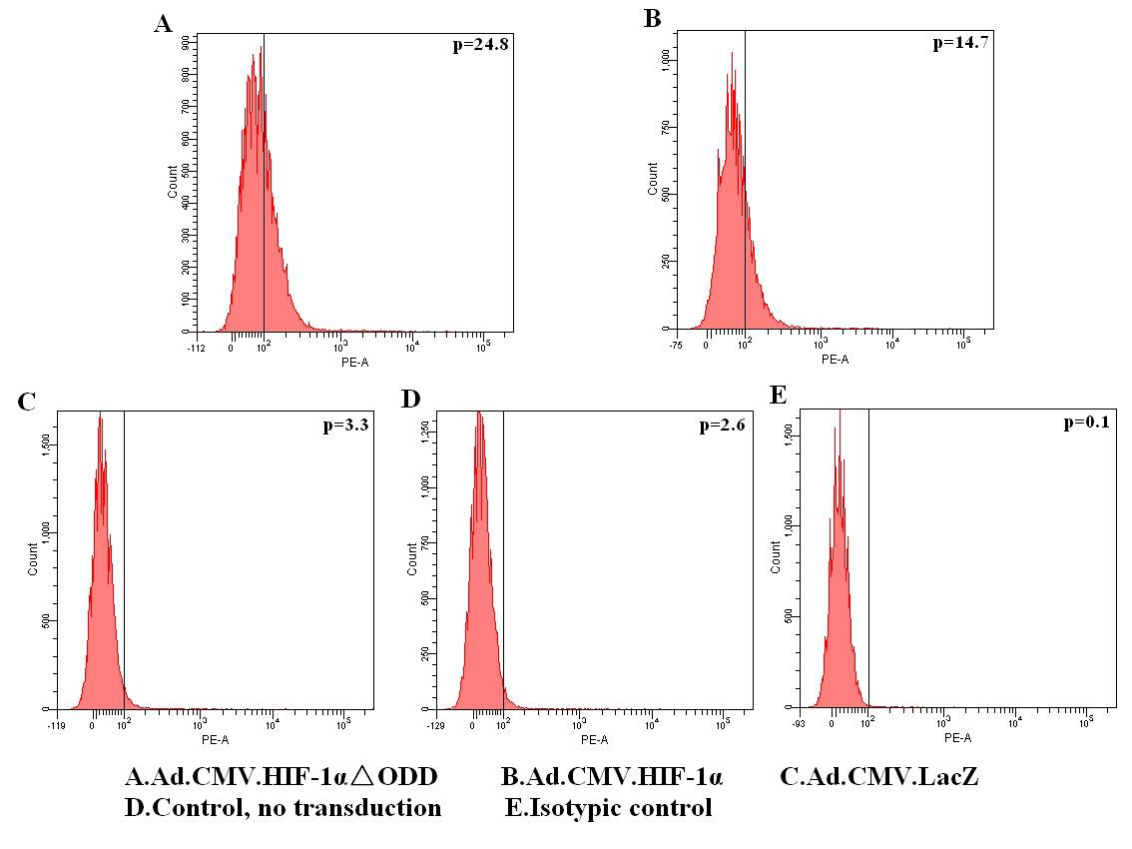
**Adenoviral mediated overexpression of HIF-1alpha and HIF-1alphaDELTAODD can up-regulate the surface expression of MICA on HK-2 cells in normoxia**. The relative protein expression levels were determined by flow cytometry with a PE conjugated mouse anti-human MICA mAb 96 h after transduction in normoxia. The percentage of cells stained by MICA mAb is shown by the numbers in the top right of each graph. (A) HK-2 transduced with Ad.CMV.HIF-1alphaDELTAODD (B) HK-2 transduced with Ad.CMV.HIF-1alpha (C) HK-2 transduced with Ad.CMV.LacZ (D) HK-2 without transduction (E) Isotype control.

As Ad.CMV.HIF-1alphaDELTAODD could mediate stable HIF-1 transcriptional activity in normoxia, we investigated whether HIF-1alpha could up-regulate MICA expression. Quantitative real-time PCR results showed that MICA mRNA levels in the groups transduced with Ad.CMV.HIF-1alphaDELTAODD or Ad.CMV.HIF-1alpha were nearly 2--4 times higher than those of the Ad.CMV.LacZ transduced and control groups 72 h after transduction, and maintained at 96 h (Figure 3). Moreover, the Ad.CMV.HIF-1alphaDELTAODD group had nearly 2-fold greater MICA mRNA expression compared with the Ad.CMV.HIF-1alpha group 72 h after transduction. These observations were also confirmed at the protein level by flow cytometry (Figure 4).

**Figure 5:**
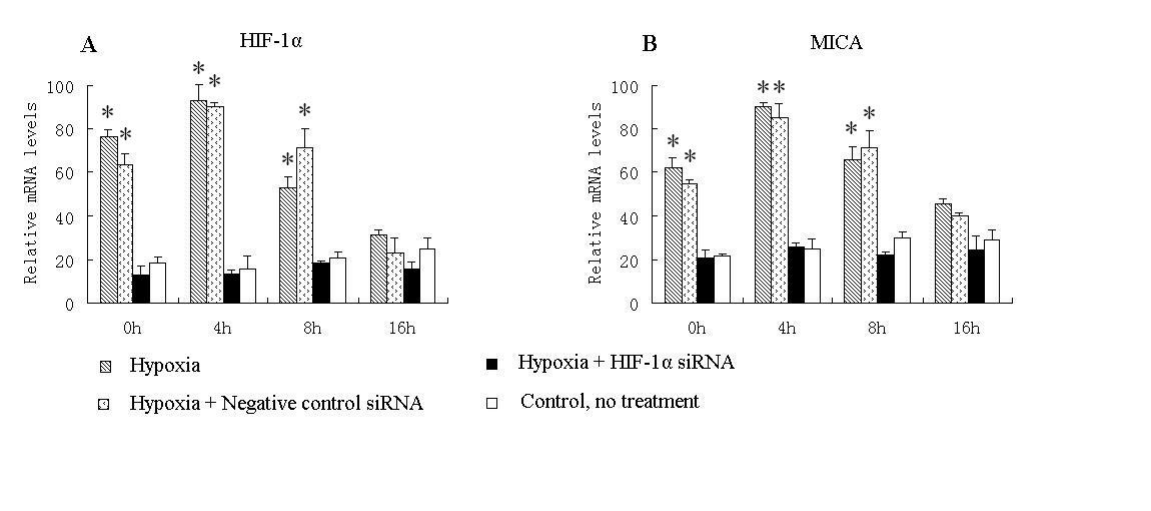
**Transduction of HIF-1alpha siRNA can inhibit HIF-1alpha and MICA expression on HK-2 cells during hypoxia/reoxygenation**. The relative mRNA levels were determined by real time PCR 0, 4, 8, and 16 h after reoxygenation and normalized to the housekeeping gene β-actin. (A) Relative mRNA levels of HIF-1alpha. (B) Relative mRNA levels of MICA. Values are shown as means ± SD from three independent experiments. *indicates a significant difference compared with the control group (p < 0.05).

**Figure 6:**
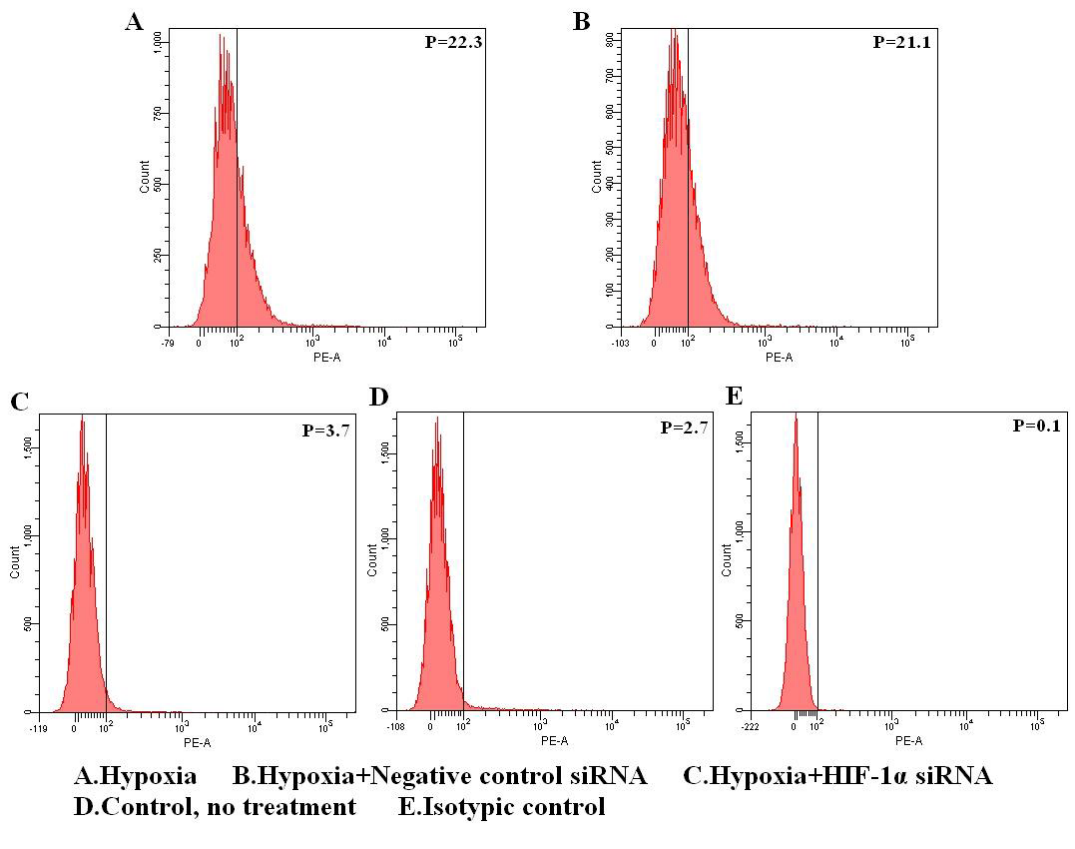
**Transduction of HIF-1alpha siRNA can inhibit surface expression of MICA on HK-2 cells during hypoxia/reoxygenation**. The relative protein expression levels were determined by flow cytometry with a PE conjugated mouse anti-human MICA mAb 8 h after reoxygenation. The percentage of cells stained by MICA mAb is shown by the numbers in the top right of each graph. (A) HK-2 cultured in hypoxia (B) HK-2 treated with negative control siRNA, cultured in hypoxia (C) HK-2 treated with HIF-1alpha siRNA, cultured in hypoxia (D) HK-2 cultured in normoxia, no treatment (E) Isotype control.

Moreover, HK-2 cells cultured in hypoxia conditions for 16 h had nearly four times higher HIF-1alpha mRNA levels immediately after reoxygenation (0h) compared with the control group (cultured in normoxia, without hypoxia/reoxygenation treatment), which increased to nearly six times 4 h after reoxygenation. This is mainly because hypoxia leads to the generation of reactive oxygen species, which can induce HIF-1alpha accumulation on reoxygenation [27,28]. However, levels decreased again after 8 h and 16 h (Figure 5A). As we expected, there is a nearly 2-fold increase in MICA mRNA expression at 0 h after reoxygenation compared with the control group, which was maintained at 4 h but decreased at 8 h and 16 h (Figure 5B). Under hypoxic conditions, RNAi treatment inhibited the increase in HIF-1alpha mRNA levels compared with the hypoxia and control groups after hypoxia/reoxygenation (Figure 5A). Correspondingly, MICA mRNA expression was barely increased (Figure 5B). Furthermore, flow cytometry analysis showed that MICA surface expression was inhibited in the RNAi treatment group compared with the hypoxia without RNAi treatment groups 8 h after reoxygenation (Figure 6).

Together, these results suggest that HIF-1 may play a very important role in up-regulating the surface expression of MICA on HK-2 cells during IRI

### HIF-1alpha expression influences NK cell cytotoxicity towards HK-2 cells

It is known that MICA triggers the cytolysis mediated by NK cells [8]. Our results above demonstrate a potential regulatory link between the MICA and HIF-1 pathways. MICA expression is up-regulated directly or indirectly by the over-expression of HIF-1alpha. However, whether HIF-1alpha over-expression could influence NK cells cytotoxicity towards HK-2 cells has not been reported. Thus, we used a commercial lactate dehydrogenase (LDH) release assay to measure the ability of NK cells to lyse HK-2 target cells.

**Figure 7:**
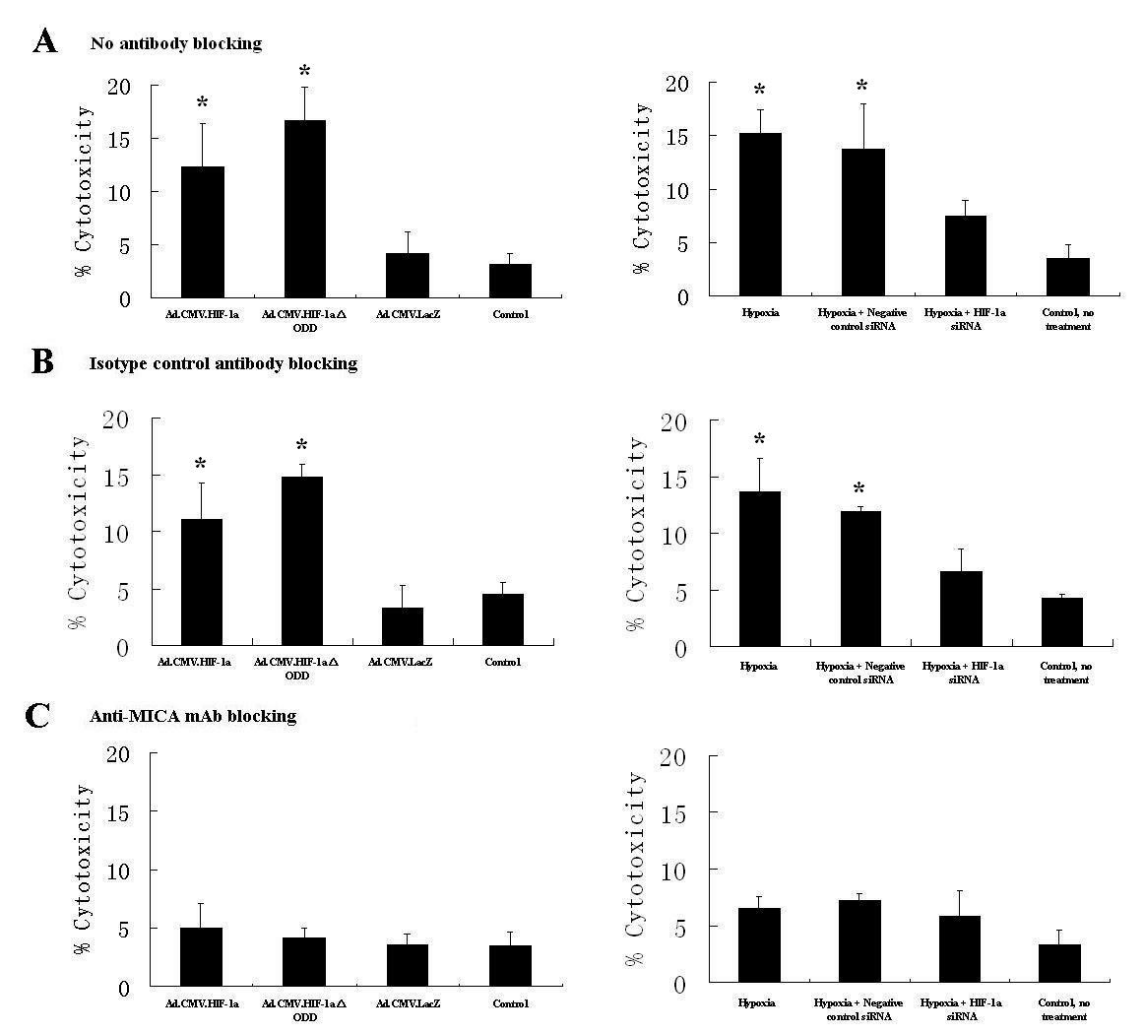
**NK cell cytotoxicity towards HK-2 cells was measured by LDH release assay kit**. HK-2 cells were collected 96 h after transduction of Ad.CMV.HIF-1alpha, Ad.CMV.HIF-1alphaDELTAODD, Ad.CMV.LacZ and 8 h after hypoxia/reoxygenation with/without RNAi treatment. Before cells were used in the cytotoxicity assay, a blocking mAb was added or not. (A) No antibody, (B) isotype-matched mAb, (C) blocking MICA mAb. LDH measurements were made in triplicate from three separate assays. Values are shown as means ± SD from three independent experiments. *indicates a significant difference compared with the control group (p < 0.05).

We found that over-expression of HIF-1alpha in normoxia causes an increase in NK cell cytotoxicity towards HK-2 cells compared with the Ad.CMV.LacZ and control groups, and that this was increased in the Ad.CMV.HIF-1alphaDELTAODD group compared with the Ad.CMV.HIF-1alpha group (Figure 7A). Moreover, RNAi-mediated inhibition of HIF-1alpha expression in hypoxia caused a decrease in NK cell cytotoxicity towards HK-2 cells compared with the hypoxia group (Figure 7A).

These results suggest that NK cell cytotoxic activity towards HK-2 cells positively correlated with HIF-1alpha expression and that MICA expression may play a role in this process.

### HIF-1alpha enhanced NK cell cytotoxicity towards HK-2 cells is due to up-regulated surface expression of MICA

To identify whether the over-expression of HIF-1alpha enhances NK cell cytotoxicity towards HK-2 cells is due to the up-regulation MICA surface expression, we used a monoclonal antibody to block this action.

Although the expression of HIF-1alpha was up-regulated by transduction of adenovirus (Ad.CMV.HIF-1alpha and Ad.CMV.HIF-1alphaDELTAODD), the blocking by anti-human MICA monoclonal antibody induced a decrease in NK cell cytotoxicity towards HK-2 cells compared with the group blocked by an isotype control antibody (Figure 7B). Moreover, blocking with MICA antibody in hypoxia in RNAi or control RNAi-treated cells caused an obvious decrease in NK cell cytotoxicity towards HK-2 cells compared with the isotype control antibody and no antibody groups (Figure 7C).

Overall, these results demonstrated that HIF-1alpha expression influences NK cell cytotoxicity towards HK-2 cells via its regulation of MICA surface expression.

### MICA expressed by HK-2 cells induces NK cell effector functions

To further demonstrate that MICA functions as a ligand of NKG2 D and that surface expression of MICA, up-regulated by HIF-1alpha, induces NK cell effector functions, we measured the IFNgamma levels in supernatants from the same cultures used in cytotoxicity assays by ELISA.

**Figure 8:**
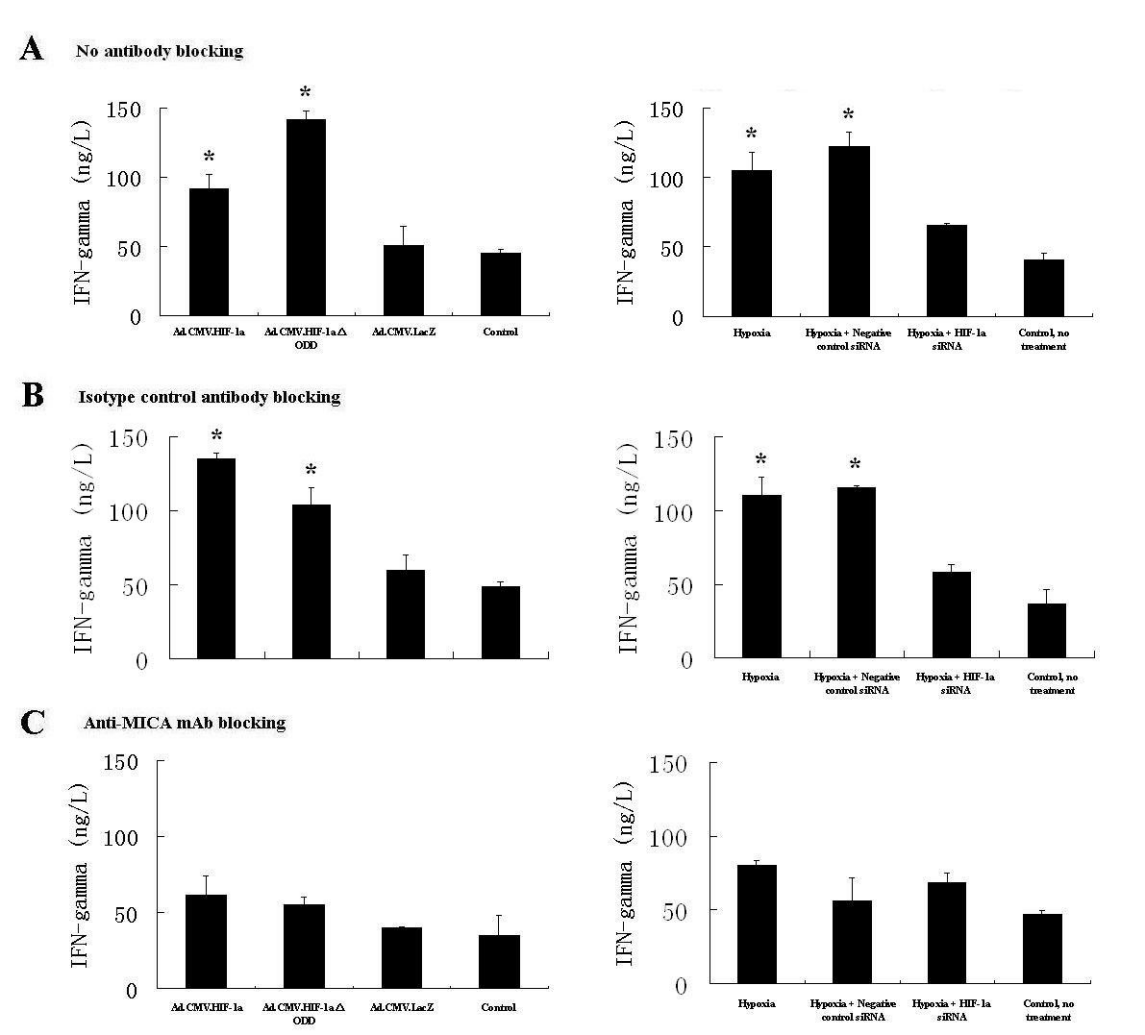
**IFNgamma levels in culture supernatants were measured by ELISA**. Culture supernatants were harvested from the same cell cultures used for cytotoxicity assays. (A) No antibody, (B) isotype-matched mAb, (C) blocking MICA mAb. IFNgamma measurements were made in triplicate from three separate assays. Values are shown as means ± SD from three independent experiments. *indicates a significant difference compared with the control group (p < 0.05).

As we expected, when the NK cell line NK92-MI was co-cultured with the HK-2 cells expressing HIF-1alpha or HIF-1alphaΔODD in normoxia, there was a 2- to 3-fold increase in IFNgamma compared with Ad.CMV.LacZ and control groups (Figure 8A). Also, inhibition of HIF-1alpha expression in hypoxia by RNAi caused a decrease in IFNgamma secretion compared with the hypoxia group (Figure 8A). To confirm that NKG2D-MICA interactions were involved in IFNgamma secretion, we also measured IFNgamma levels in supernatants from HK-2 cells that were blocked with the blocking MICA mAb or an isotype control before the cytotoxicity assays. Our results showed that the blocking MICA mAb induced a decrease in IFNgamma secretion compared with the isotype control antibody and no antibody groups (Figure 8B and Figure 8C).

These results demonstrated that HIF-1alpha over-expression induced NK cell effector functions via its regulation of MICA surface expression, strongly supporting the notion that MICA is a NKG2 D ligand.

## Discussion

In recent years, there has been an increasing interest in non-HLA antigens as mediators of injury in solid organ transplantation, due to the fact that kidney transplants also undergo immunological rejections in the absence of detectable HLA antibodies [1,2,4]. As a non-HLA antigen, MICA expressed on endothelial and epithelial cells has been implicated in the pathogenesis of hyperacute, acute and chronic organ allograft rejections. Stastny has demonstrated that MICA might be a target during anti-graft immune responses in transplanted patients [29]. In clinical studies, Sumitran-Holgersson demonstrated a close connection between the presence of MICA antibodies and early immunological responses in kidney allograft recipients, even though donor specific HLA antibodies were undetected [1]. Moreover, Ozawa reported that the presence of MICA antibodies is closely related to chronic kidney allograft rejection [3,30], while Mizutani reported that the frequency of anti-MICA antibodies is higher among recipients who undergo kidney allograft rejection compared with recipients who do not [10]. Thus, MICA plays an prominent role in kidney allograft outcome.

Zwirner has demonstrated that antibodies against MICA are often produced after transplantation [31]. In the latest review, Suarez-Alvarez mentioned that it is possible that the expression of MICA in human grafts could be induced by IRI [32], because it has been reported that IRI induces expression of retinoic acid early inducible 1 (RAE-1, MIC homolog that functions as a ligand for mouse NKG2D) on tubular epithelial cells in kidneys, contributing to acute allograft rejection [13].

In this study, we found that MICA expression was up-regulated by HIF-1alpha on human renal proximal tubular epithelial cells during hypoxia/reoxygenation. Using the HIF-1alphaDELTAODD-expressing adenovirus, HIF-1alpha was functionally and steadily expressed regardless of oxygen tension, providing a useful cell model for determining whether HIF-1alpha could influence MICA expression in normoxia. Our results showed that 96 h after transduction, Ad.CMV.HIF-1alpha and Ad.CMV.HIF-1alphaDELTAODD groups showed a substantial increase in MICA mRNA and surface expression compared with the Ad.CMV.LacZ and control groups. On the other hand, after 16 h of hypoxia and reoxygenation for 8 h, there was a decrease in MICA mRNA and surface expression levels in the HIF-1alpha siRNA treatment group compared with the hypoxia group. Taken together, these results demonstrated that the up-regulated surface expression of MICA on HK-2 cells during hypoxia/reoxygenation correlates with the over-expression of HIF-1alpha. These findings support our hypothesis that during organ IRI in kidney transplantation, the MICA surface expression on allografts is up-regulated by HIF-1alpha, inducing antibodies against MICA in the recipients' sera after transplantation, leading to poor kidney transplant outcomes [1,3,4,10,31].

NKG2 D ligands (such as MICA) are key targets of the immune response. NKG2 D is an activating receptor that is ubiquitously expressed by NK cells [6], a major component of the innate immune system [33]. Binding of NKG2 D to its ligands, such as MICA, activates NK cells and promotes cytotoxic lysis of the cells expressing these molecules [8], which might lead to graft loss. Since adenovirus mediated HIF-1alpha/HIF-1alphaDELTAODD transduction could up-regulate MICA expression, we proposed that it may enhance NK cell cytotoxicity towards target cells, which over-express HIF-1alpha/HIF-1alphaDELTAODD. As expected, NK cells exhibited greater cytotoxicity towards the Ad.CMV.HIF-1alpha and Ad.CMV.HIF-1alphaDELTAODD groups compared with the Ad.CMV.LacZ and control groups 96 h after transduction. Moreover, an inhibition of HIF-1alpha expression by siRNA showed a great decrease in NK cell cytotoxicity compared with the hypoxia without RNAi treatment group 8 h after reoxygenation. Thus, NK cell cytotoxicity towards HK-2 cells positively correlated with HIF-1alpha expression, and blocking with a MICA antibody demonstrated that it is mediated by the surface expression of MICA, which is up-regulated by HIF-1alpha over-expression. Moreover, our ELISA results also demonstrated that co-culture of NK cells and HK-2 cells expressing MICA, which was up-regulated by over-expression of HIF-1alpha, could induce IFNgamma secretion by NK cells. Interestingly, although HIF-1alpha could induce many genes that protect cells from hypoxia stress, such as HO-1 and VEGF, it also up-regulates ligands of NKG2 D, such as MICA, enhancing NK cell cytotoxicity towards target cells, leading to their destruction. More studies are needed, to understand the balance of its dual role in helpful and harmful effects. In addition, although the inhibition of HIF-1alpha could induce an decrease NK cell cytotoxicity towards target cells, only 50% is inhibited compared with the hypoxia and hypoxia with negative control RNAi treatment groups, which may not correspond to the level of inhibition seen for MICA surface expression on HK-2 cells. This indicates that there are probably other ways, in addition to the HIF-1 pathway, which could also influence NK cell cytotoxic activity towards HK-2 cells under hypoxia conditions, which would require further study.

In addition, we also found that the potential hypoxia response elements to which HIF-1 binds during hypoxia contains a core sequence 5'-CGTG-3'[34] 1000 base pairs upstream from the transcriptional start site of MICA (data not shown). However, in this study, we have not formally proved that MICA expression is up-regulated by HIF-1alpha directly or indirectly, it needs further studies because it might be important for developing strategies to reduce the harmful effect of MICA in kidney transplant outcome in the future.

## Conclusion

To summarize, our studies confirmed that HIF-1alpha plays a very important role in up-regulating MICA expression and enhancing NK cell cytotoxicity towards target cells during hypoxia/reoxygenation in HK-2 cells.

These findings provide a better understanding of MICA activation mechanisms during kidney transplantation, and a putative mechanism for the influence of IRI on transplant outcome: HIF-1alpha up-regulates the surface expression of MICA on grafts during renal IRI, which may all recognition by NK cells leading to cytotoxicity against the organ. This will lead to the production of antibodies against MICA, which can kill cells in the presence of the recipient's serum complement, leading to acute or chronic kidney allograft rejection.

## Methods

### Gene cloning, modification and vector plasmid construction

Ad.CMV.HIF-1alpha, Ad.CMV.HIF-1alphaDELTAODD and Ad.CMV.LacZ (recombinant adenovirus carrying β-galactosidase DNA, generated as a control adenoviral construct), which encode the HIF-1alpha, HIF-1alphaDELTAODD, and β-galactosidase, respectively, were constructed as described previously [35].

### Cells and culture conditions

Human renal proximal tubular epithelial cell line (HK-2) were purchased from American Type Culture Collection (ATCC, USA) and cultured in Dulbecco's modified eagle medium (DMEM) (Invitrogen, USA) with non-essential amino acids, 0.05 mg/ml bovine pituitary extract, 50 ng/ml human recombinant epidermal growth factor, 100 units/ml penicillin, 100 ug/ml streptomycin and 10% fetal bovine serum under a 5%CO_2 _and 95% air atmosphere at 37°C.

The human NK cell line (NK-92, purchased from ATCC) was cultured in alpha minimum essential modified medium (alphaMEM) without ribonucleosides and deoxyribonucleosides (Invitrogen) and supplemented with 12.5% fetal bovine serum (FBS), 12.5% horse serum, 0.2 mM inositol, 0.1 mM β2-mercaptoethanol, 0.02 mM folic acid and 100--200 U/ml recombinant IL-2 under a 5% CO_2 _and 95% air atmosphere at 37°C.

### Transduction of adenoviral vectors into HK-2 cells

Ad.CMV.HIF-1alpha, Ad.CMV.HIF-1alphaDELTAODD and Ad.CMV.LacZ were propagated in the 293A cell line and then purified by CsCl centrifugation. Titers of adenoviral stocks were measured by a standard TCID50 technique. The results ranged from 3 × 10^10 ^to 4 × 10^10 ^pfu/ml.

On the day of transduction, adenoviral stocks were added into the wells of a 6-well plate containing 80% confluent HK-2 cells at an MOI of 100 DNA particles per cell. 24 h later, the medium was replaced with fresh complete DMEM. At 24, 48, 72 and 96 h after transduction, cells were collected for the following assays.

### RNA interference (RNAi) treatment of cells

SiRNA oligonucleotides for HIF-1alpha and negative control siRNA were designed and purchased from RiboBio (China). The primer pairs were used as follows: HIF-1alpha sense (5'-UCAAGUUGCUGGUCAUCAGdTdT-3'), HIF-1alpha antisense (5'-CUGAUGACCAGCAACUUGAdTdT-3'); negative control siRNA (product.no.Ncontrol_05815, RiboBio).

On the day of RNAi treatment, the media was removed from 50% confluent HK-2 cells cultured in a 12-well plate and 800 μl Opti-Mem I (Invitrogen) was added. Then HIF-1alpha or negative control siRNA was diluted in Opti-Mem I to a final volume of 200 μl containing 2 μl Lipofectamine 2000 reagent (Invitrogen) and incubated at room temperature for 25 min before addition to each well. The final working concentration of HIF-1alpha and negative control siRNA was 100nM. 24 h later, the culture medium was replaced with RPMI medium 1640 without glucose and serum. For simulating IRI in vitro, the HK-2 cells were incubated under low-oxygen conditions (37°C, 2% O_2_, 5% CO_2 _and 93% N_2_) for 16 h, after which they were cultured in normoxic conditions (37°C, 5%CO_2 _and 95% air) for reoxygenation. At 0, 4, 8 and 16 h after reoxygenation, cells were collected for the following assays.

### RNA preparation and quantitative real time PCR analysis

After the treatments described above, HK-2 cells were harvested and total RNA was isolated using standard TRIZOL (Invitrogen) protocols. cDNA was synthesized using PrimeScript RT reagent kit (Takara, Japan) in accordance with the manufacturer's manual. Human HIF-1alpha, MICA, heme oxygenase-1 (HO-1) and vascular endothelial growth factor (VEGF) were quantified by real time PCR using SYBR Premix Ex Taq (Takara) and iCycler iQ sequence Detection System (Bio-Rad, USA). Primers for HIF-1alpha, MICA, HO-1 and VEGF were as follows: HIF-1alpha sense (5'-CAAGAACCTACTGCTAATGC-3') and HIF-1alpha antisense (5'-TTATGTATGTGGGTAGGAGATG-3'); MICA sense (5'-GTTTCTGCTGTTGCTGCTGCTGC-3') and MICA antisense (5'-ATCCCTGTGGTCACTCGTCC-3'); HO-1 sense (5'-GCCAGCAACAAAGTGCAAGA-3') and HO-1 antisense (5'-AAGGACCCATCGGAGAAGC-3'); VEGF (all isoforms) sense (5'-ACAGGGAAGAGGAGGAGATG-3') and VEGF antisense (5'-GCTGGGTTTGTCGGTGTTC-3'). The mRNA abundance of human β-actin was used as the internal standard. Primers for β-actin were sense (5'-AAGATCATTGCTCCTCCTG-3') and antisense (5'-CGTCATACTCCTGCTTGCTG-3'). For each sample, triplicate determinations were made by RNA from three separate culture wells and the identities of the PCR products were confirmed by sequencing

### Immunofluorescence Studies

HK-2 cells were plated into 12-well plates at approximately 60% confluency before immunofluoresence studies of HIF-1alpha and HIF-1alphaDELTAODD protein expression after adenovirus transduction (Ad.CMV.HIF-1alpha, Ad.CMV.HIF-1alphaDELTAODD, Ad.CMV.LacZ). The medium was replaced with fresh complete DMEM 24 h later as described above and incubated for 24, 48, 72 and 96 h. Then the cells were fixed with ice-cold 4% paraformaldehyde solution for 20 min and permeabilized in 0.2% TritonX-100 for 20 min. Cells were then blocked with 1%BSA for 30 min at room temperature. The primary mouse monoclonal anti-HIF-1alpha (1:400 dilution, immunogen human HIF-1alpha aa. 610--727, recognizes both HIF-1alpha and HIF-1alphaDELTAODD, BD Transduction Laboratories, USA) was incubated with the cells for 18 h at 4°C, and the secondary polyclonal rabbit anti-mouse immunoglobulins/FITC (1:50 dilution, DAKO, Denmark) was added to the cells for 1 h at 37°C. The cells were then observed using an Olympus 1X70 microscope. (Olympus, JP)

### Flow cytometry

MICA surface expression on HK-2 cells was analyzed by flow cytometry with a PE conjugated mouse IgG2b anti-human MICA monoclonal antibody (R&D, USA). PE conjugated mouse IgG2b isotype control was used to monitor background staining levels (R&D). Samples were collected after transduction of adenovirus or RNAi treatment and analyzed on a FACSAria cell-sorting system (BD).

### Cytotoxicity assay

NK cell cytotoxicity towards HK-2 cells was measured using the lactate dehydrogenase (LDH)-based CytoTox96non-radioactive cytotoxicity assay kit (Promega, US) in accordance with the manufacturer's protocol. LDH is a stable cytosolic enzyme that is released into the media upon cell lysis. For NK cell cytotoxicity assays, HK-2 cells were collected 96 h after transduction and 8 h after hypoxia/reoxygenation, then plated in a round bottom 96-well plate (2 × 10^4 ^cells per well). By using a 6:1 effector cell to target cell ratio, NK cells (1.2 × 10^5 ^per well) were added to the wells and the plate was then incubated at 37°C for 4 h. Cells were pelleted by centrifugation at 250 × g for 4 min. The supernatant (50 ul) was transferred to a new 96-well plate and 50 μl of reconstituted Substrate Mix was then added. The plate was incubated at room temperature for 30 min in the dark. The reaction was stopped with the addition of 50 μl Stop Solution and the absorbance was measured at 490 nm. Each condition was tested in triplicate.

### Antibody blocking experiment

HK-2 cells were collected 96 h after transduction and 8 h after hypoxia/reoxygenation and washed twice in PBS containing 5%FBS. Cells were incubated with mouse IgG1 anti-human MICA monoclonal antibody (R&D) or mouse IgG1 isotype antibody (R&D) for 2 h at 37°C, and then washed twice in fresh complete medium. After that, cytotoxicity was measured as described above.

### IFNgamma ELISA

Culture supernatants were harvested from the same cell cultures used for cytotoxicity assays. Soluble interferon gamma (IFNgamma) levels in culture supernatants were measured using enzyme-linked immunosorbent assay (ELISA) kits in accordance with the manufacturer's manual (R&D). For each sample, triplicate determinations were made in supernatants from three separate culture wells.

### Statistics

Results expressed as mean values (SD) were analyzed by a one way ANOVA analysis followed by the LSD test. A value of p < 0.05 was considered statistically significant.

## List of abbreviations

MICA: human major histocompatibility complex class I-related chain A; HIF-1: hypoxia-inducible factor-1; ODD: O_2_-dependent degradation domain; IRI: Ischemia/reperfusion injury; HK-2: Human renal proximal tubular epithelial cell line; LDH: lactate dehydrogenase; HO-1: Heme oxygenase-1; VEGF: vascular endothelial growth factor.

## Competing interests

The authors declare that they have no competing interests.

## Authors' contributions

JL and LW conceived the study, established the design and carried out the experimental work. LD and FSL performed the HK-2 and NK92 cell culture. JS participated in the gene cloning, modification and vector plasmid construction. LL and JYG contributed to the design, participated in the antibody blocking experiment, NK cytotoxicity assay and critical revision of the manuscript. PYL and PYL participated in the data analysis and provided critical comments on the study design and manuscript. LF contributed to the design and coordination of the study, and helped to draft the final version of this manuscript. All authors read and approved the final manuscript.
